# Function and Regulation of Histone H3 Lysine-4 Methylation During Oocyte Meiosis and Maternal-to-Zygotic Transition

**DOI:** 10.3389/fcell.2020.597498

**Published:** 2020-10-09

**Authors:** Qian-Qian Sha, Jue Zhang, Heng-Yu Fan

**Affiliations:** ^1^Fertility Preservation Laboratory, Reproductive Medicine Center, Guangdong Second Provincial General Hospital, Guangzhou, China; ^2^Clinical Research Center for Reproduction and Genetics in Hunan Province, Reproductive and Genetic Hospital of CITIC-Xiangya, Changsha, China; ^3^Life Sciences Institute, Zhejiang University, Hangzhou, China

**Keywords:** genome reprogramming, histone modification, histone methyl transferase, early embryo, zygote, germ cell, epigenetics

## Abstract

During oogenesis and fertilization, histone lysine methyltransferases (KMTs) and histone lysine demethylases (KDMs) tightly regulate the methylation of histone H3 on lysine-4 (H3K4me) by adding and removing methyl groups, respectively. Female germline-specific conditional knockout approaches that abolish the maternal store of target mRNAs and proteins are used to examine the functions of H3K4 KMTs and KDMs during oogenesis and early embryogenesis. In this review, we discuss the recent advances in information regarding the deposition and removal of histone H3K4 methylations, as well as their functional roles in sculpting and poising the oocytic and zygotic genomes. We start by describing the role of KMTs in establishing H3K4 methylation patterns in oocytes and the impact of H3K4 methylation on oocyte maturation and competence to undergo MZT. We then introduce the latest information regarding H3K4 demethylases that account for the dynamic changes in H3K4 modification levels during development and finish the review by specifying important unanswered questions in this research field along with promising future directions for H3K4-related epigenetic studies.

## Fertilization and Maternal-To-Zygotic Transition in Mammals

In mammals, fertilization of an oocyte by a sperm results in the generation of a totipotent embryo that can form all cell types of the embryonic and extraembryonic lineages. Before, during, and shortly after fertilization, both parental genomes are transcriptionally silent, and early embryonic events are controlled by maternal mRNAs and proteins that are stored during oocyte growth (Dai et al., 2018). Acquisition of totipotency is accompanied by the chromatin remodeling of highly differentiated parental genomes, removal of the maternal transcripts and proteins, and zygotic genome activation (ZGA) (Zhang et al., 2015; Yu et al., 2016; Sha et al., 2018b).

Before ZGA, early embryonic development is controlled exclusively by maternal products accumulated during oocyte development. After the elimination of a subset of the maternal products, transcription is re-initiated and developmental control passes to the zygotic genome (Tadros and Lipshitz, 2009). This drastic transition from a highly differentiated oocyte to a totipotent embryo is referred to as the maternal-to-zygotic transition (MZT) (Sha et al., 2019). Histone methylation is the major determinant of the formation of transcriptionally active and inactive regions of the genome and is crucial for proper chromatin remodeling during oogenesis and the MZT (Clarke and Vieux, 2015).

Gametes are highly differentiated cell types. Therefore, fertilization requires major epigenetic remodeling to reconcile the paternal and maternal genomes with the formation of a totipotent zygote (Yu et al., 2013). At the time of fertilization, the paternal genome is densely packed with protamines, and the maternal epigenome is highly specialized in terms of DNA and histone modifications. During the MZT, maternal factors unravel these specialized chromatin states to enable ZGA and embryonic development. Histone H3 lysine methylations are dynamically regulated during the MZT and have different cellular and physiological impacts, depending on the modified amino acid residue and the number of added methyl groups (mono-, di-, or tri-). However, the maternal and zygotic regulators underlying these drastic histone modifications and their impact on ZGA and embryonic development remain unknown.

## Histone Lysine Methyltransferases and Demethylases

Histone methylation is tightly regulated by histone lysine methyltransferases (KMTs) and histone lysine demethylases (KDMs), which add and remove methyl groups, respectively. KMTs and KDMs have specificities for their target lysine residues, as well as for the number of methyl groups they can add or remove. Some of these enzymes have been genetically knocked out in mice. Deletion often leads to prenatal or perinatal lethality. To overcome early lethality, selected promoter fragments of *Ddx4*, *Gdf9*, and *Zp3* were used to drive the oocyte-specific expression of CRE recombinase in the developing oocytes of transgenic mice, starting from embryonic day-16 (oocyte cyst stage) (Gallardo et al., 2007), postnatal day-3 (primordial follicle stage) (Lan et al., 2004), and day-5 (primary follicle stage) (Lewandoski et al., 1997), respectively. Using these genetic tools, the physiological functions of KMTs and KDMs during oogenesis and the MZT are being discovered.

## Histone H3 Lysine-4 Trimethylation (H3K4me3)

H3K4me3 is a common histone modification at the transcription start site of actively expressing genes in eukaryotic cells (Howe et al., 2017). Because of the association between H3K4me3 and gene expression levels, H3K4me3 is generally believed to have an instructive role in gene expression and thus is an ‘activating’ histone modification. In mouse embryonic stem cells, for example, H3K4me3 is associated with gene promoters and poises them for transcriptional activation in response to developmental or environmental cues (Blackledge and Klose, 2011). However, many recent genome-wide studies have shown that under steady-state or dynamically changing conditions, transcription actually changes very little upon removal of most H3K4me3 on the chromatin (Clouaire et al., 2014). Therefore, rather than serving as instructions for transcription, the deposition of H3K4me3 onto chromatin is suggested to be a consequence of transcription and is thought to influence processes such as transcriptional consistency among cells in a population, transcriptional memory of previous differentiation states, and gene silencing. In the following sections, we will discuss the functions and regulations of H3K4 methylation associated with transcription and organization of the mammalian genome in oocytes and zygotes, some of which are similar to those observed in somatic cells, whereas others are unique to these specific cell types.

## Function of Histone H3K4 Methyltransferases in Oogenesis and Mzt

The SET1/COMPASS histone methyltransferase complex is the primary enzyme that methylates histone H3K4 (Roguev et al., 2001). While yeast contains only one SET1 protein, there are six known histone H3 methyltransferases in mammals. They are subdivided into three groups, i.e., SET domain-containing 1A/B (SETD1A/B), lysine (K) methyltransferase 2A/B (KMT2A/B), and KMT3/4 (Shilatifard, 2012). In previous publications, KMTs were also known as mixed lineage leukemia 1-4 (MLL1-4); however, these are no longer the official gene names on NCBI. These complexes are not functionally redundant, as demonstrated by the early lethality phenotypes observed upon the knockout of individual genes (Vedadi et al., 2017). These studies also showed that SETD1A/B-based enzyme complexes are the predominant H3K4 methyltransferases in most cell types (Ardehali et al., 2011; Shilatifard, 2012).

### CxxC–Finger Protein-1 (CXXC1)

The SETD1 complex targets chromatin by its essential subunit CxxC-finger protein 1 (CXXC1, also known as CFP1), which recognizes both preexisting H3K4me3 and non-methylated DNA and engages in multivalent chromatin binding of the whole complex (Lee and Skalnik, 2005; Brown et al., 2017).

During meiotic prophase, a diploid germ cell produces haploid gametes with two consecutive rounds of division. Unique chromosomal events occur during the prophase of meiosis I, including programmed double-strand breaks and genome-wide homologous recombination (Borde and de Massy, 2013). Successful homologous recombination and crossover formation are essential for the precise chromosomal segregation and fertility (de Massy, 2013). Studies in yeasts have shown that CXXC1 recruits chromatin regions with H3K4me3 to the chromosome axis for DNA double-strand break generation and crossover formation in the prophase of meiosis (Parvanov et al., 2017). To investigate whether CXXC1 also plays a comparable role in the meiotic prophase of mammalian germ cells, researchers from two groups independently knocked out *Cxxc1* in germ cells before the onset of meiosis with *Stra8-Cre* (Tian et al., 2018; Jiang et al., 2020). While the knockout of *Cxxc1* in male germ cells using transgenic *Stra8-Cre* did not affect spermatogenesis and male fertility, the deletion of *Cxxc1* in a *Stra8-Cre* knockin mouse strain resulted in male and female infertility. In the male mice of this line, spermatogenesis is arrested at metaphase II (MII). Phenotype analysis results showed that CXXC1 is essential for proper meiotic crossover formation. The deletion of *Cxxc1* causes a decrease in H3K4me3 levels from the pachytene to the MII stage and gene transcription disorder. These studies suggest that CXXC1-mediated H3K4me3 not only directly controls meiosis-specific chromatin behaviors as in yeast but also plays an essential role in regulating genes essential for the meiotic progression of spermatogenesis and oogenesis.

The meiosis-related function of CXXC1 is more extensively investigated in postnatal female mice in which the oocytes undergo arrest at the diplotene stage of meiotic prophase I. Studies in oocyte-specific *Cxxc1* knockout mice indicated that SETD1-CXXC1 is one of the major KMT complexes that mediate H3K4me3 deposition on chromatin in mouse oocytes (Yu et al., 2017). Conditional knockout of *Cxxc1* in growing oocytes results in defects in histone exchanges, DNA methylation, and transcription of the oocytic genome. Furthermore, decreases in maternal H3K4me3 impaired *de novo* histone deposition during pronuclear formation after fertilization and prevented ZGA.

In addition to MZT defects, meiotic resumption and spindle assembly are impaired in fully grown *Cxxc1*-deleted oocytes (Yu et al., 2017; Sha et al., 2018a). The involvement of CXXC1 and H3K4me3 in the regulation of meiotic cell cycle progression is discussed in later sections.

### SET Domain-Containing 1A and B (SETD1A/B)

*Setd1a* and *Setd1b* are highly conserved paralogs. During development, *Setd1a* and *Setd1b* are expressed at all stages from the oocyte to the blastocyst stage (Bledau et al., 2014). The zygotic deletion of *Setd1a* or *Setd1b* does not affect preimplantation (Bledau et al., 2014). *Setd1a* is required shortly after inner cell mass formation because *Setd1a* null embryos die soon after implantation [embryonic day (E) 6.5–7.5], and no embryonic stem cell lines could be generated using the *Setd1a* null embryos. In contrast, *Setd1b* null embryos are growth retarded from E7.5 and die around E11.5 (Bledau et al., 2014).

To overcome embryonic lethality, oocyte-specific *Setd1a* and *Setd1b* knockout mouse strains (*Setd1a^*fl/fl*^; Gdf9-Cre* and *Setd1b^*fl/fl*^; Gdf9-Cre*) were generated and analyzed (Bledau et al., 2014; Brici et al., 2017). *Setd1a* deletion in the developing oocytes did not perturb fertility. However, in *Setd1b^*fl/fl*^; Gdf9-Cre* females, follicular loss accumulated with age. The ovulated MII oocytes exhibited abnormalities associated with the meiotic spindle. *Setd1b* null oocytes and zygotes displayed perturbed cytoplasmic organelles and aggregated lipid droplets. Even if the maternal *Setd1b*-null oocytes were fertilized, most zygotes underwent an arrest at the pronuclear stage and displayed polyspermy in the perivitelline space. None of these zygotes develop beyond the 4-cell stage (Brici et al., 2017). In many aspects, these phenotypes mimic those observed in maternal *Cxxc1* knockout oocytes and embryos, and reinforce the statement that SETD1-CXXC1 methyltransferase is essential for enhancing the developmental competence of mouse oocytes.

However, a serious concern is raised that no differences in the global levels of H3K4 methylation, including H3K4me1, H3K4me2 or H3K4me3, were observed between the control and *Setd1b* null oocytes and zygotes (Brici et al., 2017). This is in sharp contrast to the *Cxxc1* null oocytes, in which the H3K4me3 level decreases significantly (Yu et al., 2017; Sha et al., 2018a). Therefore, these results suggest two facts, i.e., (Dai et al., 2018) SETD1A and SETD1B play overlapping roles in mediating H3K4 trimethylation during oogenesis, whereas CXXC1 is indispensable as a DNA-binding subunit of the SETD1/CAMPASS complex, and (Sha et al., 2018b) the absence of SETD1B may affect the distribution— instead of the global abundance—of H3K4m3 in the maternal genome and cause milder defects than the *Cxxc1* knockout in oocytes.

### Lysine Methyltransferase 2B (KMT2B)

KMT2B, also known as MLL2, activates gene expression by mediating the tri-methylation of histone H3 lysine 4 at the promoters of genes involved in embryogenesis and hematopoiesis (Glaser et al., 2006). KMT2B is a large protein composed of approximately 2700 amino acids that is cleaved by the taspase 1 threonine endopeptidase to give rise to N- and C-terminal fragments; both of which are subunits of the functional KMT2B/COMPASS complex. KMT2B-N, KMT2B-C, WDR5, RBBP5, and ASH2L serve as the core catalytic component of the KMT2B/COMPASS complex, which is recruited to target genes.

The deletion of *Kmt2b* in mouse oocytes using *Gdf9-Cre* results in anovulation, oocyte death, and female infertility (Andreu-Vieyra et al., 2010). Oocyte-specific knockout of *Kmt2b* leads to decreased H3K4me3 levels, abnormal meiotic maturation, and gene expression. In addition, ZGA is compromised in the absence of *Kmt2b*. Knockout of *Kmt2b* in oocytes results in the loss of transcription-independent ncH3K4me3 but has relatively moderate influences on transcription-coupled H3K4me3 accumulation or gene expression (Hanna et al., 2018). Together these results indicate that KMT2B is another key H3K4 methyltransferase in the epigenetic reprogramming required for oogenesis and MZT in mouse.

**FIGURE 1:**
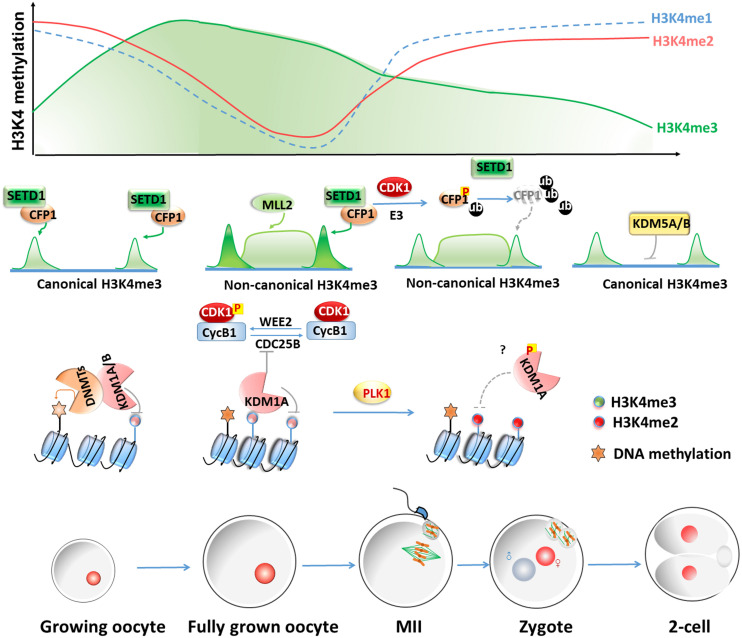
Dynamic changes and regulations of methylated histone H3 levels during oocyte-to-embryo transitions in mouse. In growing oocytes, H3K4me3 remains a canonical pattern of narrow peaks at the gene promoters. The deposition of the non-canonical form of H3K4me3 (ncH3K4me3) coincides with genome silencing from fully grown oocytes to the early two-cell stage, which exists as broad peaks at promoters and a large number of distal loci. NcH3K4me3 is deposited over intergenic regions and distal elements independently of transcription through the action of KMT2B during oocyte maturation. During meiotic maturation, CDK1 triggers both CXXC1 (CFP1) phosphorylation and degradation. The degradation of CXXC1 is a robust way to remove the SETD1-CXXC1 complex from chromatin and results in the decrease of H3K4me3 levels. KDM5B is responsible for actively removing broad H3K4me3 domains and resetting the canonical H3K4me3 peaks until the two-cell stage. KDM1A and KDM1B catalyze H3K4me1 and H3K4me2 demethylations and have crucial roles in mammalian oogenesis and the MZT. KDM1B is required for proper DNA methylation at imprinted DMR in the growing oocyte. In the fully grown oocytes, KDM1A is essential for maintaining meiotic arrest by inhibiting the upregulation of the CDK1 phosphatase CDC25B. KDM1A phosphorylated by PLK1 dissociates from chromatin during mitosis. It is worth testing if this regulation also exists in oocytes and causes an increase of H3K4me2 level during oocyte maturation.

However, it should be noted that KMT2B is the only important KMT2 family H3K4 methyltransferase in oocytes because the potential functions of KMT2A, KMT2C, and KMT2D have not been investigated in oocytes. It will be interesting to test whether these KMT2 family members are also involved in regulating oogenesis and MZT in mammalian species using conditional gene knockout approaches. The dynamic changes and regulations of methylated histone H3 during oocyte-to-embryo transition in mouse is summarized in Figure 1.

## Role of H3K4me3 in Meiotic Cell Division

Despite extensive studies on H3K4me3 in the context of transcription-related cellular events, the direct function of H3K4me3 with respect to chromatin behavior during cell division has been elusive because of the absence of H3K4me3 in most cell types affects the transcription of a broad spectrum of house-keeping genes and causes cell cycle arrest in the G1 or S phase. Considering these technical difficulties, a fully grown mammalian oocyte is an ideal model to study transcription-independent functions of epigenetic modifications because *de novo* gene transcription is neither required nor active during the two sequential meiotic divisions.

In the ovaries of female mammals, all oocytes within the pre-antral follicles have a non-surrounded nucleolus (NSN) type of chromatin configuration. The chromatin of fully grown germinal vesicle oocytes undergoes a transformation from the NSN to the surrounding nucleolus (SN) type when the follicles grow to the antral stage (Tan et al., 2009). SN type oocytes have better developmental competence after fertilization than NSN oocytes (Ma et al., 2013; Zhang et al., 2019). In developing mouse oocytes, H3K4me3 levels on chromatin increase during the transition of chromatin configuration from the NSN to SN type (Yu et al., 2017). Following meiosis resumption, H3K4me1 and H3K4me2 levels increase, but H3K4me3 levels decrease after GV breakdown (GVBD) and reach the lowest point in anaphase I (Sha et al., 2018a). The meiotic maturation-coupled fluctuation of H3K4 methylation levels suggested that these histone modifications perform previously unrecognized transcription-independent functions with respect to regulating the meiotic divisions of oocytes.

When *Gdf9-Cre* was employed to knock out *Cxxc1* in oocytes as early as the primordial follicle stage, the *Cxxc1*-null oocytes exhibited reduced GVBD and polar body 1 (PB1) emission rates during meiotic maturation than wild type oocytes (Sha et al., 2018a). They also fail to assemble bipolar spindles because chromosomes are not able to align at the equatorial plates of the meiotic spindles. CXXC1 is likely to be directly involved in these processes because the expression of a dominant-negative CXXC1 mutant using mRNA microinjection in fully grown oocytes leads to defects similar to those observed in *Cxxc1* null oocytes. Because the genome of the fully grown mammalian oocytes does not have transcriptional activities, these results suggest that CXXC1-mediated H3K4 trimethylation might have a transcription-independent role during mouse oocyte meiotic maturation.

The phosphorylation of histone H3 at threonine-3 (H3T3ph) during the G2-M transition is required for both mitotic and meiotic divisions (Wang et al., 2012, 2016). CXXC1-dependent H3K4 trimethylation is a permissive signal for the subsequent H3T3 phosphorylation (Sha et al., 2018a), but the exact mechanism is unclear. Hypothetically, a decreased H3K4me3 level in oocytes results in chromatin tightening. As a result, haspin, a kinase that triggers H3T3 phosphorylation, cannot easily access the chromatin during meiosis resumption. The interplay between K4 methylation and T3 phosphorylation in histone H3 has also been investigated using biochemical studies. Analysis of the haspin crystal structure revealed that the bulkiness of methylated K4 prevents the interaction of the H3 tail with the narrow substrate-binding groove of haspin (Eswaran et al., 2009). *In vitro* kinase assay results indicate that K4 methylation impairs the T3 phosphorylation of histone H3 by haspin (Eswaran et al., 2009). Furthermore, H3T3ph is located adjacent to—while not overlapping with—H3K4me3 on the chromosomes of mouse oocytes (Sha et al., 2018a). All these studies indicate that H3K4me3 regulates T3 phosphorylation in an intermolecular manner. These hierarchical histone modifications on the maternal genome are involved in the precise regulation of meiotic cell cycle progression.

## Cell Cycle-Coupled Phosphorylation and Degradation of CXXC1

**FIGURE 2:**
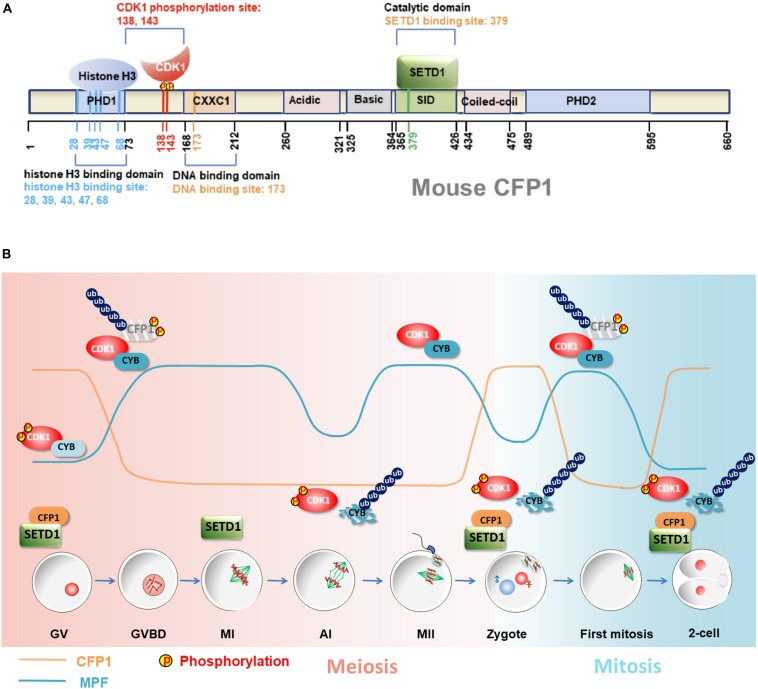
Role of CXXC1-SETD1 H3K4 methyltransferases in oocyte development and MZT. **(A)** A schematic representation of mouse CXXC1 (CFP1). Important CXXC1-binding proteins and their binding sites are shown. The amino acid positions of distinct functional domains are indicated. **(B)** The dynamic changes of CDK1 activity (blue line) and CXXC1 protein level (orange line) in oocytes and early embryos. CDK1 activity is inhibited by dephosphorylation at Thr-14 and Tyr-15 during the G-M transition and is activated by Thr-14/Tyr15 dephosphorylation as well as cyclin B binding. Activated CDK1 triggers CXXC1 phosphorylation and degradation following the meiotic resumption. CXXC1 proteins are reaccumulated until exiting meiosis and are degraded repeatedly in the following mitotic cell cycles in association with CDK1 activation.

CXXC1 proteins are rapidly degraded in oocytes after meiotic resumption, remain undetectable at the MII stage, and reaccumulate after fertilization (Sha et al., 2018a). The same study has shown that CXXC1 degradation is mediated by 65 amino acids present at the C-terminal. As a cell cycle-coupled regulator of CXXC1, CDK1 directly interacts with CXXC1 and phosphorylates it at two conserved consensus CDK1 target sites (Ser-138 and Ser-143) during the G2–M transition and triggers CXXC1 degradation. The ubiquitin E3 ligase that mediates CXXC1 degradation has not yet been identified. CDK1 may trigger CXXC1 degradation by modulating the activity of this E3 ligase or its accessibility to CXXC1 during the G2-M transition. In addition, phosphorylation of CXXC1 at Ser-138 and Ser-143 impairs its binding to histone H3, thereby inhibiting the ability of CXXC1 to mediate H3K4 trimethylation (Sha et al., 2018a) (Figure 2A).

CXXC1 depletion during meiotic division is of physiological importance. Chromatin undergoes extensive reorganization during oocyte meiosis and fertilization, transitioning from a relatively relaxed configuration during maturation to a highly condensed state in mature eggs and returning to the interphase state after fertilization (Zhang et al., 2015). The degradation of CXXC1 is a robust means of ensuring the absence of SETD1-CXXC1 complex from the chromatin during this transition, thereby facilitating chromosome condensation. If the chromosome-bound CXXC1 proteins are not degraded in maturing oocytes, they can hinder chromosomal condensation during meiotic spindle assembly. Overexpression of stabilized CXXC1 (C-terminus-deleted CXXC1) in fully grown GV oocytes results in decreased GVBD and PB1 emission rates, as well as defects in spindle assembly. Furthermore, these phenotypes become more prominent upon additional deletions of CXXC1 phosphorylation sites (Sha et al., 2018a) (Figure 2B).

## H3K4me3 and Replacement of Histone Variants During MZT

There are three variants of histone H3 in mammals, namely H3.1, H3.2, and H3.3. H3.1 and H3.2 variants are deposited on chromatin during the S phase of the cell cycle because their deposition is DNA replication-dependent. In contrast, the H3.3 variant is expressed and deposited in chromatin throughout the cell cycle in a DNA replication-independent manner. Dynamic histone exchanges in the chromatin of the oocytic and zygotic genomes are essential for maintaining normal transcriptional activity. Particularly, histone modifications associated with active gene expression, such as H3K4me3, are enriched in H3.3. Accordingly, the deposition of H3.3 is correlated with transcriptionally active genes.

Akiyama et al. (2011) examined the global deposition of histone H3 variants after fertilization in mice. H3.1 and H3.3 occupy unusual heterochromatic and euchromatin locations, whereas H3.2 is incorporated into the transcriptionally silent heterochromatin. Maternal H3.3 protein is incorporated into the paternal genome as early as 2 h post-fertilization, and is detectable in the paternal genome until the morula stage (Kong et al., 2018). The knockdown of maternal H3.3 results in compromised transcription from the paternal genome after fertilization as well as embryonic development (Wen et al., 2014). Collectively, these findings indicate that active changes in the deposition of histone H3 variants are critical for chromatin reorganization during MZT.

Related to the focus of this review, the replacement of H3 variants is also regulated by post-translational modifications of H3. H3.3 is enriched in H3K4me3, whereas H3.1 is enriched in dimethylated H3 lysine-9 (H3K9me2). In a histone replacement experiment—involving microinjection of mRNAs encoding Flag-tagged histone H3 variants into GV stage-arrested *Cxxc1*-null oocytes—the H3.3 incorporation rates were significantly decreased when compared with those in the control oocytes. The newly translated H3.3 proteins underwent remarkable *de novo* lysine-4 trimethylation in wild type oocytes in a CXXC1-dependent manner, even when DNA replication and transcription activities were absent. Furthermore, DNase I digestion assay for assessing chromatin accessibility in oocytes revealed that the genomic DNA of CXXC1-deleted oocytes was more resistant to DNase I digestion than the DNA from wild type oocytes. These results indicate that SETD1-CXXC1-mediated H3K4 trimethylation is essential for maintaining proper chromatin configurations, which makes the genome accessible to protein factors that facilitate transcription during oocyte development.

## Asymmetrical Distribution of Methylated H3K4 in Pronuclei After Fertilization

In mammals, the maternal and paternal genomes are not functionally equivalent; both the maternal and paternal genomes are required for embryonic development. The most distinguishing feature that differentiates the sperm genome from the oocyte genome is that it is globally compacted with protamine proteins rather than with histone proteins (Feil, 2009). However, the protamines are promptly removed after fertilization and are replaced by maternal histones stored in the oocytes. Unlike the maternal chromatin, which maintains all types of histone H3 methylations throughout embryonic development, paternal chromosomes acquire new and unmodified histones during or after the formation of the male pronucleus.

Shortly after the formation of the male and female pronuclei, H3K4me1 exhibits comparable levels in both pronuclei; however, H3K4me2 and H3K4me3 are initially present at lower levels in male pronuclei than in the female pronuclei. The acquisition of H3K4me2/me3 in the male pronucleus only occurs at the latest pronuclear stages (Lepikhov and Walter, 2004). Researchers have logically assumed that the H3K4me3 modifications in the female pronuclei are inherited from those that were deposited on the maternal chromosomes during oogenesis. However, a recent study indicated that this might not be so simple (Yu et al., 2017).

The *Cxxc1*-deleted oocytes can be fertilized, but none of the embryos developed beyond the 2-cell stage, indicating that the absence of maternal CXXC1 in oocytes affects the developmental potential of the resulting zygotes after fertilization (Yu et al., 2017); these zygotes exhibited severe defects in ZGA. In a rescue experiment—involving the microinjection of mRNAs encoding CXXC1 into the maternal *Cxxc1*-deleted zygotes—CXXC1 reaccumulated in both male as well as female pronuclei. Surprisingly, H3K4me3 levels were restored only in the female but not in male pronuclei (Yu et al., 2017). This asymmetrical H3K4 trimethylation in rescued zygotes that mimic wild type zygotes, but reveals two previously unknown facts, i.e., (Dai et al., 2018) the H3K4me3 modification in female pronuclei are not only inherited from the maternal chromosome but are also generated *de novo* after fertilization, and (Sha et al., 2018b) H3K4me3 modification is incapable of being generated in male pronuclei, even in the presence of CXXC1. Further investigations, such as the localization of SETD1A/B and chromosome accessibility to CXXC1 in male pronuclei, are needed to understand the mechanisms underlying this phenomenon.

Consistent with the essential role of the H3K4me3 modification in ZGA, maternal HIRA—a chaperone for the histone variant H3.3 mediating its chromosomal deposition—is required for zygote development in mice (Lin et al., 2014; Nashun et al., 2015). The formation of the male pronucleus is inhibited upon the knockout of maternal *Hira* owing to the absence of the nucleosome assembly in the paternal genome after fertilization (Lin et al., 2014). Because H3K4 trimethylation occurs predominantly on H3.3, the absence of maternal HIRA may result in zygotic developmental arrest by impairing H3K4me3 generation in the pronuclei. Furthermore, *Hira*-null oocytes fail to develop parthenogenetically, indicating a role for HIRA in the female pronuclei (Lin et al., 2014).

## Function of Histone H3K4 Demethylases in Oogenesis and Fertilization

### K Demethylase 1A

KDM1A, also known as lysine-specific demethylase 1 (LSD1), specifically catalyzes the demethylation of H3K4me1 and H3K4me2. Mammals contain two members of the KDM1 family, KDM1A and KDM1B (also known as LSD2). Both enzymes play crucial roles in mammalian oogenesis and MZT (Kim et al., 2015; Ancelin et al., 2016). KDM1A is widely expressed in multiple somatic tissues during development, whereas KDM1B is specifically expressed in growing mouse oocytes (Ciccone et al., 2009).

Genetic deletion of *Kdm1a* prior to gastrulation results in early lethality (Metzger et al., 2005). In developing oocytes, KDM1A balances global H3K4me2 levels and is essential for female fertility. *Kdm1a*-deficient oocytes exhibit defects in maintaining prophase I arrest and undergo precocious GVBD, partially due to the upregulation of CDK1 phosphatase CDC25B (Kim et al., 2015). *Kdm1a*-null oocytes also exhibit derepression of retrotransposons, which increases genome instability and DNA damage as well as spindle and chromosomal defects that cause aneuploidy. The majority of *Kdm1a*-null oocytes undergo apoptosis before the completion of meiotic maturation (Kim et al., 2015). Another group reported that knockout of maternal *Kdm1a* causes embryonic developmental arrest at the 2-cell stage, accompanied by dramatic alterations in genomic H3K4 methylation patterns (Ancelin et al., 2016). Furthermore, the absence of maternal KDM1A results in the derepression of the LINE-1 retrotransposon in the resulting embryos and increases genome instability. Therefore, maternal KDM1A plays a critical role in establishing appropriate H3K4 methylation patterns in the zygote during MZT.

In a manner similar to CXXC1, KDM1A is also regulated by cell cycle-coupled phosphorylation in cultured human cell lines. Mitosis kinase polo-like kinase-1 (PLK1) directly interacts with KDM1A and phosphorylates it at Serine-126 (Peng et al., 2017). As a result, phosphorylated KDM1A dissociates from chromatin during mitosis, but the cellular effects of KDM1A dissociation from chromatin are unclear. It is worthwhile to test if this regulation also exists in oocytes and early embryos, and plays a functional role in meiotic maturation and blastomere cleavage.

### K Demethylase 1B (KDM1B)

Differential DNA methylation of the paternal and maternal alleles—known as differentially methylated regions (DMRs)—regulates the parental origin-specific expression of imprinted genes in mammalian genomes. Epigenetic imprints in male and female germ cells are established during gametogenesis and are maintained throughout development. The *de novo* DNA methyltransferase (DNMT) 3A and its cofactor DNMT3-like (DNMT3L) play a direct role in this process (Ooi et al., 2007). In addition, histone H3K4 methylation also plays a role in regulating germline imprinting.

KDM1B is highly expressed in growing oocytes and is required for the *de novo* DNA methylation in some imprinted genes in the oocytes (Ciccone et al., 2009). The deletion of *Kdm1b* in mice does not affect embryo development, animal survival, or oocyte growth. However, *Kdm1b*-null oocytes from the conditional knockout females show abnormally high levels of H3K4 methylation and fail to deposit DNA methylation marks at many maternal-imprinted loci. Early embryos derived from these maternal *Kdm1b*-null oocytes show biallelic expression or suppression of the affected genes and fail to develop beyond the mid-gestation stage (Ciccone et al., 2009). This phenotype is reminiscent of the *Dnmt3a* or *Dnmt3l* knockout mice, which also have a maternal imprinting defect in the oocytes.

KDM1B is an H3K4me2-specific histone demethylase. Dramatic changes in bulk H3K4me2 were observed in *Kdm1b*-null oocytes, suggesting that KDM1B acts on a large proportion of histone H3 in the chromatin. However, *Kdm1b* knockout precisely affects DNA methylation at the DMRs of specific imprinted loci, instead of global DNA methylation in maturing oocytes. The reason why *Kdm1b* deficiency selectively affects some, but not all, maternally imprinted genes remains to be explained. A follow-up study shows that maternal genomic regions destined for DNA methylation in oocytes of both primary and growing follicles exhibit reduced H3K4me2/3 levels (Stewart et al., 2015). Overall, these results indicate that H3K4 methylation has a protective function against—or restricts—*de novo* DNA methylation during oocyte development. A plausible explanation for this function is that the demethylation of H3K4 facilitates the access of *de novo* DNA methylation machinery to the destined maternal DNA imprinted loci. This hypothesis is supported by the observation that DNMT3L interacts with histone H3, and methylation at H3K4 strongly inhibits this interaction (Otani et al., 2009).

### K Demethylase 5B (KDM5B)

There are two major classes of KDMs, i.e., the KDM1 subfamily and the KDM2-KDM7 subfamily, which contain a Jumonji C (JmjC) domain (Xhabija and Kidder, 2018). The KDM5 family includes KDM5A-D (Xhabija and Kidder, 2018) and plays important roles in regulating H3K4 methylation by catalyzing the demethylation processes. Knockout of *Kdm5b* resulted in early embryonic lethality, whereas mice not expressing *Kdm5a* are viable and fertile (Catchpole et al., 2011), suggesting that *Kdm5b* is the major functional KDM family member *in vivo*. In a study investigating the transcriptome defects that may account for the developmental arrest of somatic cell nuclear transfer (SCNT) embryos at the 4-cell stage, Liu et al. (2016) observed that *Kdm5b* fails to be activated in 4-cell stage-arrested SCNT embryos. To determine the role of *Kdm5b* in the development of SCNT embryos, they deleted or overexpressed *Kdm5b* in SCNT embryos, and found that the developmental potential of these embryos was decreased and increased, respectively (Liu et al., 2016). Co-injection of mRNAs encoding KDM5B and KDM4B (a H3K9me3 demethylase) can restore transcriptional profiles and improve the blastocyst development rate of SCNT embryos (Liu et al., 2016). These results suggest that KDM5B is an important epigenetic factor involved in genome reprogramming in SCNT embryos.

In the normal ZGA process, *Kdm5a* and *Kdm5b* show peak expression in 2-cell embryos and are responsible for actively removing broad H3K4me3 domains. Embryos depleted for *Kdm5a*/*b* retain high levels of H3K4me3 at the late 2-cell stage. A significant number of ZGA genes were downregulated in *Kdm5a/b*-depleted 2-cell embryos. As a result, the *Kdm5a/b*-depleted embryos fail to develop to the blastocyst stage (Dahl et al., 2016). Therefore, both the formation of broad H3K4me3 domains during oogenesis and the KDM5A/B-mediated timely H3K4me3 demethylation at the 2-cell stage are crucial for MZT. However, an oocyte-specific *Kdm5b* knockout mouse model has not been established and investigated (Table 1). The potentially important role of KDM5B in oogenesis and MZT needs to be confirmed by future *in vivo* studies.

**TABLE 1:** Role of H3K4 methyltransferases and demethylases in oocyte development and MZT.

Protein (*Gene*)	Biochemical activity	Phenotype of KO	Phenotype of cKO in oocyte	Phenotype of maternal KO in zygote	References
SETD1A (*Setd1a*)	H3K4 Lysine methyl- transferase (H3K4me3)	Fail to gastrulate and die at E7.5	No phenotype	No phenotype	Bledau et al., 2014
SETD1B (*Setd1b*)	H3K4 Lysine methyl- transferase (H3K4me3)	Growth retardation from E7.5; die at E11.5	Follicular loss with age; Ovulated MII oocytes exhibited abnormalities of the zona pellucida and meiotic spindle.	*Setd1b*-null oocytes can be fertilized, but none of these zygotes develop beyond the 4-cell stage.	Bledau et al., 2014; Brici et al., 2017
CXXC1 (*Cxxc1*)	DNA/histone-binding subunit of SETD1 complex (H3K4me3)	Die between E6.5 and E12.5	Compromised histone exchanges, DNA methylation, transcription of the oocytic genome, and spindle assembly.	Maternal *Cxxc1* KO impairs the MZT after fertilization and *de novo* histone deposition. Embryos arrest at the 1- or 2-cell stage.	Carlone and Skalnik, 2001; Yu et al., 2017; Sha et al., 2018a
KMT2B^1^	H3K4 Lysine methyl-transferase non-canonical (nc) H3K4me3	Embryonic failure before E11.5	*Kmt2b* knockout in oocytes results in anovulation and oocyte death.	ZGA is compromised, and embryos arrest between the 1-cell and 2-cell stages.	Glaser et al., 2006; Andreu-Vieyra et al., 2010; Hanna et al., 2018
KDM1A (*Kdm1a*)	H3K4 Lysine demethylase (H3K4me2)	Early lethality prior to gastrulation.	Arrest at prophase I due to the upregulation of the CDK1 phosphatase CDC25B.	Impairs the MZT and arrests at the 2-cell stage.	Kim et al., 2015; Ancelin et al., 2016
KDM1B (*Kdm1b*)	H3K4 Lysine demethylase (H3K4me2)	Viable	Fail to establish maternal DNA methylation imprints.	No MZT defects. Embryos from *Kdm1b*-null oocytes die before mid-gestation.	Ciccone et al., 2009
KDM5B (*Kdm5b*)	H3K4 Lysine demethylase (H3K4me3)	Early embryonic lethality before E7.5	Overexpression of KDM5B reactivates transcription in surrounded nucleolus oocytes.	Phenotype of cKO was no reported. Knockdown of KDM5A/B in zygotes impairs ZGA at the 2-cell stage.	Catchpole et al., 2011; Dahl et al., 2016

*^1^Also known as MLL2.*

## Perspectives

After fertilization, the maternal and paternal genomes form haploid female and male pronuclei, respectively. These two separate nuclei coexist in the zygote. How these two epigenetically distinct genomes are temporally and spatially reorganized following the initiation of embryo development is poorly understood. Recently, several groups have developed single-nucleus high-resolution chromosome conformation capture methods that provide greater sensitivities than the previous methods to investigate three-dimensional chromatin organization in rare cell types, including the oocytes, zygotes, and blastomeres of early embryos (Du et al., 2017; Ke et al., 2017). They showed that the chromatin organization of germ cells and zygotes is fundamentally different from that of somatic cells. Oocytes display distinct features of genomic organization, but these chromatin architectures are uniquely reorganized during the MZT. Understanding the role of H3K4 modification in these processes could potentially provide insights into reprogramming differentiated cells to a totipotent state.

Accumulating evidence indicates that H3K4me3 is essential for ZGA, but the underlying mechanisms are not adequately addressed. The timing of the transcription initiation after fertilization (known as minor ZGA) is species-specific and occurs at the mid-1-cell stage in mice (Abe et al., 2018). A low level of enhancer-independent transcription occurs promiscuously in a large proportion of genomic regions during minor ZGA. The expression pattern of these genes is very different from that at later stages (Abe et al., 2015). Generally, the chromatin structure is repressive for transcription, and enhancers are required to help transcription factors access the gene promoters. In zygotes, however, transcriptional activity is not stimulated by enhancers. A plausible hypothesis suggests that in zygotes with an extremely loose chromatin structure, transcription factors can easily access the DNA and cause promiscuous gene expression (Yamamoto and Aoki, 2017). The role of H3K4me3 in this working model remains to be elucidated.

Lastly, the findings described above indicate that the spatiotemporal-specific establishment and erasure of H3K4me3 during oocyte development enables the oocyte genome to establish the competence to generate a healthy embryo in a cell-autonomous manner. Nevertheless, the extent to which this epigenetic modification forms a cell non-autonomous instructive component of ovarian follicle development remains unclear. Recent studies have revealed that appropriate levels of H3K4me3 accumulation in growing oocytes are necessary to maintain the expression of genes encoding oocyte-derived paracrine factors, including growth and differentiation factor 9 (GDF9), bone morphogenic protein 15 (BMP15), and fibroblast growth factor 8 (FGF8) (Sha et al., 2020). CXXC1-dependent expression of these genes facilitates communication between an oocyte and the surrounding ovarian somatic cells and is required for the establishment of distinct gene expression patterns in granulosa cells and cumulus cells. Oocytes that have high levels of H3K4me3 have greater potential to support follicle development to the ovulation stage, whereas follicles containing oocytes with low H3K4me3 levels are prone to undergo atresia before ovulation. Future investigations are required to elucidate the cell non-autonomous role of H3K4me3 in germ cells and blastomeres of preimplantation embryos.

## Author Contributions

H-YF and Q-QS conceived and wrote the manuscript. JZ prepared the figures. All the authors contributed to the article and approved the submitted version.

## Conflict of Interest

The authors declare that the research was conducted in the absence of any commercial or financial relationships that could be construed as a potential conflict of interest.

## References

[B1] Abe K. Yamamoto R. Franke V. Cao M. Suzuki Y. Suzuki M. G. (2015). The first murine zygotic transcription is promiscuous and uncoupled from splicing and 3’ processing. *EMBO J.* 34 1523–1537. 10.15252/embj.201490648 25896510 PMC4474528

[B2] Abe K. I. Funaya S. Tsukioka D. Kawamura M. Suzuki Y. Suzuki M. G. (2018). Minor zygotic gene activation is essential for mouse preimplantation development. *Proc. Natl. Acad. Sci. U.S.A.* 115 E6780–E6788. 29967139 10.1073/pnas.1804309115 PMC6055165

[B3] Akiyama T. Suzuki O. Matsuda J. Aoki F. (2011). Dynamic replacement of histone H3 variants reprograms epigenetic marks in early mouse embryos. *PLoS Genet.* 7:e1002279. 10.1371/journal.pgen.1002279 21998593 PMC3188537

[B4] Ancelin K. Syx L. Borensztein M. Ranisavljevic N. Vassilev I. Briseno-Roa L. (2016). Maternal LSD1/KDM1A is an essential regulator of chromatin and transcription landscapes during zygotic genome activation. *eLife* 5:e08851. 10.7554/eLife.08851 PMC4829419 26836306

[B5] Andreu-Vieyra C. V. Chen R. Agno J. E. Glaser S. Anastassiadis K. Stewart A. F. (2010). MLL2 is required in oocytes for bulk histone 3 lysine 4 trimethylation and transcriptional silencing. *PLoS Biol.* 8:e1000453. 10.1371/journal.pbio.1000453 20808952 PMC2923083

[B6] Ardehali M. B. Mei A. Zobeck K. L. Caron M. Lis J. T. Kusch T. (2011). *Drosophila* Set1 is the major histone H3 lysine 4 trimethyltransferase with role in transcription. *EMBO J.* 30 2817–2828. 10.1038/emboj.2011.194 21694722 PMC3160253

[B7] Blackledge N. P. Klose R. (2011). CpG island chromatin: a platform for gene regulation. *Epigenetics* 6 147–152. 10.4161/epi.6.2.13640 20935486 PMC3278783

[B8] Bledau A. S. Schmidt K. Neumann K. Hill U. Ciotta G. Gupta A. (2014). The H3K4 methyltransferase Setd1a is first required at the epiblast stage, whereas Setd1b becomes essential after gastrulation. *Development* 141 1022–1035. 10.1242/dev.098152 24550110

[B9] Borde V. de Massy B. (2013). Programmed induction of DNA double strand breaks during meiosis: setting up communication between DNA and the chromosome structure. *Curr. Opin. Genet. Dev.* 23 147–155. 10.1016/j.gde.2012.12.002 23313097

[B10] Brici D. Zhang Q. Reinhardt S. Dahl A. Hartmann H. Schmidt K. (2017). Setd1b, encoding a histone 3 lysine 4 methyltransferase, is a maternal effect gene required for the oogenic gene expression program. *Development* 144 2606–2617. 10.1242/dev.143347 28619824

[B11] Brown D. A. Di Cerbo V. Feldmann A. Ahn J. Ito S. Blackledge N. P. (2017). The SET1 complex selects actively transcribed target genes via multivalent interaction with CpG island chromatin. *Cell Rep.* 20 2313–2327. 10.1016/j.celrep.2017.08.030 28877467 PMC5603731

[B12] Carlone D. L. Skalnik D. G. (2001). CpG binding protein is crucial for early embryonic development. *Mol. Cell. Biol.* 21 7601–7606. 10.1128/mcb.21.22.7601-7606.2001 11604496 PMC99931

[B13] Catchpole S. Spencer-Dene B. Hall D. Santangelo S. Rosewell I. Guenatri M. (2011). PLU-1/JARID1B/KDM5B is required for embryonic survival and contributes to cell proliferation in the mammary gland and in ER+ breast cancer cells. *Int. J. Oncol.* 38 1267–1277. 21369698 10.3892/ijo.2011.956

[B14] Ciccone D. N. Su H. Hevi S. Gay F. Lei H. Bajko J. (2009). KDM1B is a histone H3K4 demethylase required to establish maternal genomic imprints. *Nature* 461 415–418. 10.1038/nature08315 19727073

[B15] Clarke H. J. Vieux K. F. (2015). Epigenetic inheritance through the female germ-line: the known, the unknown, and the possible. *Semin. Cell Dev. Biol.* 43 106–116. 10.1016/j.semcdb.2015.07.003 26183189

[B16] Clouaire T. Webb S. Bird A. (2014). Cfp1 is required for gene expression-dependent H3K4 trimethylation and H3K9 acetylation in embryonic stem cells. *Genome Biol.* 15:451 10.1186/preaccept-8577431391252814 PMC4189735 25201068

[B17] Dahl J. A. Jung I. Aanes H. Greggains G. D. Manaf A. Lerdrup M. (2016). Broad histone H3K4me3 domains in mouse oocytes modulate maternal-to-zygotic transition. *Nature* 537 548–552. 10.1038/nature19360 27626377 PMC6283663

[B18] Dai X. X. Jiang J. C. Sha Q. Q. Jiang Y. Ou X. H. Fan H. Y. (2018). A combinatorial code for mRNA 3’-UTR-mediated translational control in the mouse oocyte. *Nucleic Acids Res.* 47 328–340. 10.1093/nar/gky971 30335155 PMC6326793

[B19] de Massy B. (2013). Initiation of meiotic recombination: how and where? Conservation and specificities among eukaryotes. *Annu. Rev. Genet.* 47 563–599. 10.1146/annurev-genet-110711-155423 24050176

[B20] Du Z. Zheng H. Huang B. Ma R. Wu J. Zhang X. (2017). Allelic reprogramming of 3D chromatin architecture during early mammalian development. *Nature* 547 232–235. 10.1038/nature23263 28703188

[B21] Eswaran J. Patnaik D. Filippakopoulos P. Wang F. Stein R. L. Murray J. W. (2009). Structure and functional characterization of the atypical human kinase haspin. *Proc. Natl. Acad. Sci. U.S.A.* 106 20198–20203. 10.1073/pnas.0901989106 19918057 PMC2777956

[B22] Feil R. (2009). Epigenetic asymmetry in the zygote and mammalian development. *Int. J. Dev. Biol.* 53 191–201. 10.1387/ijdb.082654rf 19378254

[B23] Gallardo T. Shirley L. John G. B. Castrillon D. H. (2007). Generation of a germ cell-specific mouse transgenic Cre line, Vasa-Cre. *Genesis* 45 413–417. 10.1002/dvg.20310 17551945 PMC2597027

[B24] Glaser S. Schaft J. Lubitz S. Vintersten K. van der Hoeven F. Tufteland K. R. (2006). Multiple epigenetic maintenance factors implicated by the loss of Mll2 in mouse development. *Development* 133 1423–1432. 10.1242/dev.02302 16540515

[B25] Hanna C. W. Taudt A. Huang J. Gahurova L. Kranz A. Andrews S. (2018). MLL2 conveys transcription-independent H3K4 trimethylation in oocytes. *Nat. Struct. Mol. Biol.* 25 73–82. 10.1038/s41594-017-0013-5 29323282

[B26] Howe F. S. Fischl H. Murray S. C. Mellor J. (2017). Is H3K4me3 instructive for transcription activation? *Bioessays* 39 1–12. 10.1002/bies.201600095 28004446

[B27] Jiang Y. Zhang H. Y. Lin Z. Zhu Y. Z. Yu C. Sha Q. Q. (2020). CXXC finger protein 1-mediated histone H3 lysine-4 trimethylation is essential for proper meiotic crossover formation in mice. *Development* 147:dev183764. 10.1242/dev.183764 32094118

[B28] Ke Y. Xu Y. Chen X. Feng S. Liu Z. Sun Y. (2017). 3D chromatin structures of mature gametes and structural reprogramming during mammalian embryogenesis. *Cell* 170 367–381.e20. 28709003 10.1016/j.cell.2017.06.029

[B29] Kim J. Singh A. K. Takata Y. Lin K. Shen J. Lu Y. (2015). LSD1 is essential for oocyte meiotic progression by regulating CDC25B expression in mice. *Nat. Commun.* 6:10116. 10.1038/ncomms10116 PMC4686821 26626423

[B30] Kong Q. Banaszynski L. A. Geng F. Zhang X. Zhang J. Zhang H. (2018). Histone variant H3.3-mediated chromatin remodeling is essential for paternal genome activation in mouse preimplantation embryos. *J. Biol. Chem.* 293 3829–3838. 10.1074/jbc.ra117.001150 29358330 PMC5846143

[B31] Lan Z. J. Xu X. Cooney A. J. (2004). Differential oocyte-specific expression of Cre recombinase activity in GDF-9-iCre, Zp3cre, and Msx2Cre transgenic mice. *Biol. Reprod.* 71 1469–1474. 10.1095/biolreprod.104.031757 15215191

[B32] Lee J. H. Skalnik D. G. (2005). CpG-binding protein (CXXC finger protein 1) is a component of the mammalian Set1 histone H3-Lys4 methyltransferase complex, the analogue of the yeast Set1/COMPASS complex. *J. Biol. Chem.* 280 41725–41731. 10.1074/jbc.m508312200 16253997

[B33] Lepikhov K. Walter J. (2004). Differential dynamics of histone H3 methylation at positions K4 and K9 in the mouse zygote. *BMC Dev. Biol.* 4:12. 10.1186/1471-213X-4-12 15383155 PMC521682

[B34] Lewandoski M. Wassarman K. M. Martin G. R. (1997). Zp3-cre, a transgenic mouse line for the activation or inactivation of loxP-flanked target genes specifically in the female germ line. *Curr. Biol.* 7 148–151. 10.1016/s0960-9822(06)00059-5 9016703

[B35] Lin C. J. Koh F. M. Wong P. Conti M. Ramalho-Santos M. (2014). Hira-mediated H3.3 incorporation is required for DNA replication and ribosomal RNA transcription in the mouse zygote. *Dev. Cell* 30 268–279. 10.1016/j.devcel.2014.06.022 25087892 PMC4134436

[B36] Liu W. Liu X. Wang C. Gao Y. Gao R. Kou X. (2016). Identification of key factors conquering developmental arrest of somatic cell cloned embryos by combining embryo biopsy and single-cell sequencing. *Cell Discov.* 2:16010. 10.1038/celldisc.2016.10 PMC4897595 27462457

[B37] Ma J. Y. Li M. Luo Y. B. Song S. Tian D. Yang J. (2013). Maternal factors required for oocyte developmental competence in mice: transcriptome analysis of non-surrounded nucleolus (NSN) and surrounded nucleolus (SN) oocytes. *Cell Cycle* 12 1928–1938. 10.4161/cc.24991 23673344 PMC3735707

[B38] Metzger E. Wissmann M. Yin N. Muller J. M. Schneider R. Peters A. H. (2005). LSD1 demethylates repressive histone marks to promote androgen-receptor-dependent transcription. *Nature* 437 436–439. 10.1038/nature04020 16079795

[B39] Nashun B. Hill P. W. Smallwood S. A. Dharmalingam G. Amouroux R. Clark S. J. (2015). Continuous histone replacement by Hira is essential for normal transcriptional regulation and de novo DNA methylation during mouse oogenesis. *Mol. Cell* 60 611–625. 10.1016/j.molcel.2015.10.010 26549683 PMC4672152

[B40] Ooi S. K. Qiu C. Bernstein E. Li K. Jia D. Yang Z. (2007). DNMT3L connects unmethylated lysine 4 of histone H3 to de novo methylation of DNA. *Nature* 448 714–717. 10.1038/nature05987 17687327 PMC2650820

[B41] Otani J. Nankumo T. Arita K. Inamoto S. Ariyoshi M. Shirakawa M. (2009). Structural basis for recognition of H3K4 methylation status by the DNA methyltransferase 3A ATRX-DNMT3-DNMT3L domain. *EMBO Rep.* 10 1235–1241. 10.1038/embor.2009.218 19834512 PMC2775176

[B42] Parvanov E. D. Tian H. Billings T. Saxl R. L. Spruce C. Aithal R. (2017). PRDM9 interactions with other proteins provide a link between recombination hotspots and the chromosomal axis in meiosis. *Mol. Biol. Cell* 28 488–499. 10.1091/mbc.e16-09-0686 27932493 PMC5341731

[B43] Peng B. Shi R. Jiang W. Ding Y. H. Dong M. Q. Zhu W. G. (2017). Phosphorylation of LSD1 by PLK1 promotes its chromatin release during mitosis. *Cell Biosci.* 7:15. 10.1186/s13578-017-0142-x PMC5364692 28344766

[B44] Roguev A. Schaft D. Shevchenko A. Pijnappel W. W. Wilm M. Aasland R. (2001). The *Saccharomyces cerevisiae* Set1 complex includes an Ash2 homologue and methylates histone 3 lysine 4. *EMBO J.* 20 7137–7148. 10.1093/emboj/20.24.7137 11742990 PMC125774

[B45] Peng B. Shi R. Jiang W. Ding Y. H. Dong M. Q. Zhu W. G. (2017). Phosphorylation of LSD1 by PLK1 promotes its chromatin release during mitosis. *Cell Biosci.* 7:15. 10.1186/s13578-017-0142-x PMC5364692 28344766

[B46] Sha Q. Q. Yu J. L. Guo J. X. Dai X. X. Jiang J. C. Zhang Y. L. (2018b). CNOT6L couples the selective degradation of maternal transcripts to meiotic cell cycle progression in mouse oocyte. *EMBO J.* 37:e99333. 10.15252/embj.201899333 PMC6293276 30478191

[B47] Sha Q. Q. Jiang Y. Yu C. Xiang Y. Dai X. X. Jiang J. C. (2020). CFP1-dependent histone H3K4 trimethylation in murine oocytes facilitates ovarian follicle recruitment and ovulation in a cell-nonautonomous manner. *Cell. Mol. Life Sci.* 77 2997–3012. 10.1007/s00018-019-03322-y 31676962 PMC11104893

[B48] Sha Q. Q. Zhang J. Fan H. Y. (2019). A story of birth and death: mRNA translation and clearance at the onset of maternal-to-zygotic transition in mammalsdagger. *Biol. Reprod.* 101 579–590. 10.1093/biolre/ioz012 30715134

[B49] Shilatifard A. (2012). The COMPASS family of histone H3K4 methylases: mechanisms of regulation in development and disease pathogenesis. *Annu. Rev. Biochem.* 81 65–95. 10.1146/annurev-biochem-051710-134100 22663077 PMC4010150

[B50] Stewart K. R. Veselovska L. Kim J. Huang J. Saadeh H. Tomizawa S. (2015). Dynamic changes in histone modifications precede de novo DNA methylation in oocytes. *Genes Dev.* 29 2449–2462. 10.1101/gad.271353.115 26584620 PMC4691949

[B51] Tadros W. Lipshitz H. D. (2009). The maternal-to-zygotic transition: a play in two acts. *Development* 136 3033–3042. 10.1242/dev.033183 19700615

[B52] Tan J. H. Wang H. L. Sun X. S. Liu Y. Sui H. S. Zhang J. (2009). Chromatin configurations in the germinal vesicle of mammalian oocytes. *Mol. Hum. Reprod.* 15 1–9. 10.1016/j.ydbio.2006.01.008 19019837

[B53] Tian H. Billings T. Petkov P. M. (2018). CXXC1 is not essential for normal DNA double-strand break formation and meiotic recombination in mouse. *PLoS Genet.* 14:e1007657. 10.1371/journal.pgen.1007657 30365547 PMC6221362

[B54] Vedadi M. Blazer L. Eram M. S. Barsyte-Lovejoy D. Arrowsmith C. H. Hajian T. (2017). Targeting human SET1/MLL family of proteins. *Protein Sci.* 26 662–676. 10.1002/pro.3129 28160335 PMC5368065

[B55] Wang F. Ulyanova N. P. Daum J. R. Patnaik D. Kateneva A. V. Gorbsky G. J. (2012). Haspin inhibitors reveal centromeric functions of Aurora B in chromosome segregation. *J. Cell Biol.* 199 251–268. 10.1083/jcb.201205106 23071152 PMC3471242

[B56] Wang Q. Wei H. Du J. Cao Y. Zhang N. Liu X. (2016). H3 Thr3 phosphorylation is crucial for meiotic resumption and anaphase onset in oocyte meiosis. *Cell Cycle* 15 213–224. 10.1080/15384101.2015.1121330 26636626 PMC4825905

[B57] Wen D. Banaszynski L. A. Liu Y. Geng F. Noh K. M. Xiang J. (2014). Histone variant H3.3 is an essential maternal factor for oocyte reprogramming. *Proc. Natl. Acad. Sci. U.S.A.* 111 7325–7330. 10.1073/pnas.1406389111 24799717 PMC4034224

[B58] Xhabija B. Kidder B. L. (2018). KDM5B is a master regulator of the H3K4-methylome in stem cells, development and cancer. *Semin. Cancer Biol.* 57 79–85. 10.1016/j.semcancer.2018.11.001 30448242 PMC6522339

[B59] Yamamoto R. Aoki F. (2017). A unique mechanism regulating gene expression in 1-cell embryos. *J. Reprod. Dev.* 63 9–11. 10.1262/jrd.2016-133 27867162 PMC5320424

[B60] Yu C. Fan X. Sha Q. Q. Wang H. H. Li B. T. Dai X. X. (2017). CFP1 regulates histone H3K4 trimethylation and developmental potential in mouse oocytes. *Cell Rep.* 20 1161–1172. 10.1016/j.celrep.2017.07.011 28768200

[B61] Yu C. Ji S. Y. Sha Q. Q. Dang Y. Zhou J. J. Zhang Y. L. (2016). BTG4 is a meiotic cell cycle-coupled maternal-zygotic-transition licensing factor in oocytes. *Nat. Struct. Mol. Biol.* 23 387–394. 10.1038/nsmb.3204 27065194

[B62] Yu C. Zhang Y. L. Pan W. W. Li X. M. Wang Z. W. Ge Z. J. (2013). CRL4 complex regulates mammalian oocyte survival and reprogramming by activation of TET proteins. *Science* 342 1518–1521. 10.1126/science.1244587 24357321

[B63] Zhang J. Zhang Y. L. Zhao L. W. Guo J. X. Yu J. L. Ji S. Y. (2019). Mammalian nucleolar protein DCAF13 is essential for ovarian follicle maintenance and oocyte growth by mediating rRNA processing. *Cell Death Differ.* 26 1251–1266. 10.1038/s41418-018-0203-7 30283081 PMC6748096

[B64] Zhang Y. L. Liu X. M. Ji S. Y. Sha Q. Q. Zhang J. Fan H. Y. (2015). ERK1/2 activities are dispensable for oocyte growth but are required for meiotic maturation and pronuclear formation in mouse. *J. Genet. Genomics* 42 477–485. 10.1016/j.jgg.2015.07.004 26408092

